# ANGPTL8 in metabolic homeostasis: more friend than foe?

**DOI:** 10.1098/rsob.210106

**Published:** 2021-09-29

**Authors:** Chang Guo, Chenxi Wang, Xia Deng, Jianqiang He, Ling Yang, Guoyue Yuan

**Affiliations:** ^1^ Department of Nephrology, Affiliated Hospital of Jiangsu University, 438 Jiefang Road, Zhenjiang 212001, Jiangsu, People's Republic of China; ^2^ Department of Endocrinology, Affiliated Hospital of Jiangsu University, 438 Jiefang Road, Zhenjiang 212001, Jiangsu, People's Republic of China

**Keywords:** ANGPTL8, betatrophin, T2DM, glucose metabolism, lipid metabolism

## Abstract

ANGPTL8 is an important cytokine, which is significantly increased in type 2 diabetes mellitus (T2DM), obesity and metabolic syndrome. Many studies have shown that ANGPTL8 can be used as a bio-marker of these metabolic disorders related diseases, and the baseline ANGPTL8 level has also been found to be positively correlated with retinopathy and all-cause mortality in patients with T2DM. This may be related to the inhibition of lipoprotein lipase activity and the reduction of circulating triglyceride (TG) clearance by ANGPTL8. Consistently, inhibition of ANGPTL8 seems to prevent or improve atherosclerosis. However, it is puzzling that ANGPTL8 seems to have a directing function for TG uptake in peripheral tissues; that is, ANGPTL8 specifically enhances the reserve and buffering function of white adipose tissue, which may alleviate the ectopic lipid accumulation to a certain extent. Furthermore, ANGPTL8 can improve insulin sensitivity and inhibit hepatic glucose production. These contradictory results lead to different opinions on the role of ANGPTL8 in metabolic disorders. In this paper, the correlation between ANGPTL8 and metabolic diseases, the regulation of ANGPTL8 and the physiological role of ANGPTL8 in the process of glucose and lipid metabolism were summarized, and the physiological/pathological significance of ANGPTL8 in the process of metabolic disorder was discussed.

## Graphical abstract

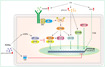

## 1. Introduction

ANGPTL8, also known as Betatrophin, RIFL, Lipasin or TD26, is a secreted protein encoded by human C19orf80 gene (mouse Gm6484 gene), is a new member of the ANGPTLs protein family [1]. Most members of ANGPTLs have similar protein structures, including a signal sequence at the N-terminus, a coiled-coil domain, and a fibrinogen/angiopoietin-like domain. Compared with other ANGPTLs, ANGPTL8 lacks the fibrinogen/angiopoietin-like domain [2]. In mice, ANGPTL8 was mainly expressed in adipose tissue and liver. In humans, its main expression site is liver, but subsequent studies also found relative expression in adipose tissue [3]. The expression of ANGPTL8 was mainly regulated by fasting/refeeding signal; that is, the concentration of circulating ANGPTL8 decreased under fasting state, while increased significantly during refeeding [4]. In addition to nutritional signals, insulin, GLP-1, vitamin D, thyroid hormones and fatty acids are also considered to be related to the regulation of ANGPTL8 expression [5–8]. In accordance with this, circulating ANGPTL8 levels also showed specific changes in a variety of metabolic diseases, including diabetes, obesity, non-alcoholic fatty liver disease (NAFLD), metabolic syndrome, polycystic ovary syndrome (PCOS) and so on [9–13].

The changes of circulating levels of ANGPTL8 in metabolic disorders, including T2DM, imply its potential value in the diagnosis of these diseases. At the same time, other studies have also explored the therapeutic value of ANGPTL8 in these diseases, which is related to its importance in regulating glucose and lipid metabolism. In previous studies, the inhibitory effect of ANGPTL3 and ANGPTL4 on LPL activity has been confirmed, which is related to specific epitope 1 (SE1) located in N-terminal domain [14,15]. Also, the role of ANGPTL8 as a ‘metabolic switch’ to regulate ANGPTL3 and ANGPTL4 on LPL inhibition differentially has recently attracted extensive attention [16]. In summary, the expressions of ANGPTL8 and ANGPTL3 were upregulated, while the expressions of ANGPTL4 were downregulated during (re)feeding. ANGPTL8 formed complexes with the other two respectively, and differentially regulated the LPL activity of adipose tissue and other oxidizing tissues, so that postprandial TG was directed into adipose tissue for storage. In the fasting state, the opposite expression regulation occurs, so that other oxidized tissues can obtain sufficient energy substrate sources [17]. Besides the regulation of lipid metabolism, several recent studies in animals have found that the effect of ANGPTL8 on improving insulin sensitivity [18]. The results of clinical prospective study also showed that the baseline ANGPTL8 level was negatively correlated with the risk of metabolic syndrome, even after multivariable adjustment [19]. Paradoxically, the results of another prospective study showed that the baseline ANGPTL8 level was positively correlated with the risk of retinopathy and all-cause mortality in patients with T2DM [20–22]. Similarly, a protein truncation variant (PTV) of ANGPTL8 has been shown to reduce the incidence of coronary artery disease (CAD) in a Finnish population [23], while another study has shown a significant reduction in cardiovascular events in CAD patients with high ANGPTL8 levels [24]. These conflicting results have led to confusion about the role of ANGPTL8 in these metabolic disorders. Therefore, it is necessary to conduct a comprehensive analysis on the regulation, physiological and pathological functions of ANGPTL8, so as to infer the role of ANGPTL8 in metabolic disorders related diseases.

## 2. Relationships between Angptl8 and metabolic diseases

### 2.1. Diabetes mellitus

In a number of independent clinical cross-sectional studies, the researchers found that circulating ANGPTL8 was significantly increased in patients with T2DM [25,26]. Previous studies of our group also found that circulating ANGPTL8 in newly diagnosed T2DM patients without drug treatment was significantly higher than that in normal glucose tolerance population, which provided stronger evidence for the correlation between ANGPTL8 and T2DM [9]. Although a small number of studies have reached inconsistent conclusions, that is, ANGPTL8 is not associated with T2DM or decreases in T2DM [27,28], generally speaking, most of the follow-up studies support the conclusion that ANGPTL8 increases in T2DM [29–32]. In this regard, some researchers explained that the ELISA kits used in these studies were different, targeting at the N-terminal or C-terminal of ANGPTL8 protein respectively, and the hydrolysis of ANGPTL8 protein may lead to different results detected by different types of kits [33,34]. Moreover, more detailed data analysis showed that ANGPTL8 was significantly correlated with insulin resistance [9,29], fasting blood glucose [35], postprandial blood glucose [36] and glycosylated haemoglobin (HbA1c) [37], which theoretically supported the association between ANGPTL8 and T2DM. In addition, the increase of ANGPTL8 was also found in prediabetes [38] and gestational diabetes mellitus (GDM) [39]. In T1DM, whether the circulating ANGPTL8 increases is still controversial [40,41]. In 2014, Espes *et al*. used ELISA to detect circulating ANGPTL8 concentrations in 33 patients with long-course T1DM and 24 age-matched healthy subjects, and found that circulating ANGPTL8 concentrations in patients with T1DM were approximately twice as high as those in healthy subjects [40]. Similarly, a clinical study from Japan involving 34 patients with T1DM and 12 healthy subjects got similar results [42]. The kits used in both studies were the same (Wuhan Eiaab Science, Wuhan, China; Catalogue No. E11644 h). However, an animal study has given the opposite result. Li *et al*. found that although circulating ANGPTL8 as well as mRNA in liver, WAT and brown adipose tissue (BAT) were significantly increased in T2DM models (diet-induced-obesity (DIO) mice and db/db mice), circulating ANGPTL8 and mRNA in tissues of T1DM models (C57B6 mice receiving streptozotocin (STZ) and 70% pancreatectomy to destroy or remove β-cells) were decreased, respectively [41].

### 2.2. Obesity

In obese patients, the circulation level of ANGPTL8 also increased significantly [25,43]. Considering the high association between obesity and T2DM (abnormal lipid metabolism, insulin resistance, etc.), this result seems to be predictable. In health, obesity, T2DM and other populations, the correlation between ANGPTL8 and a variety of lipid metabolism indicators has been fully proved. It is very clear that there is a significant positive correlation between ANGPTL8 and circulating TG levels [44]. This is due to the fact that ANGPTL8 has a direct regulatory effect on LPL activity, which we will discuss in detail below. In addition to TG, different studies have suggested the correlation between ANGPTL8 and TC, LDL and HDL, indicating that there is an inseparable relationship between ANGPTL8 and lipid metabolism, as well as diseases characterized by disorder of lipid metabolism [45,46].

### 2.3 Non-alcoholic fatty liver disease

A study by Cengiz *et al*. involving 69 patients with NAFLD diagnosed by liver biopsy and 69 healthy controls showed that the circulating ANGPTL8 in patients with NAFLD was significantly lower than that in healthy subjects, and the higher the degree of liver fibrosis, the lower the ANGPTL8 level [47]. However, Lee *et al*. showed the opposite results; that is, circulating ANGPTL8 in patients with NAFLD was higher than that in healthy people. In addition, they detected that the expression level of ANGPTL8 in liver of mice with NAFLD, including db/db or ob/ob mice and mice with a high-fat or methionine-choline deficient diet, was also increased, and considered to be related to endoplasmic reticulum stress [48]. Several follow-up clinical studies basically supported the results of Lee *et al*. [49,50].

### 2.4. Coronary artery disease

Hyperlipidaemia can lead to atherosclerosis, which greatly increases the incidence of CAD and peripheral artery disease (PAD). Circulating ANGPTL8 levels were also increased in CAD and PAD patients [51,52]. Animal studies have shown that ANGPTL8 accelerates atherosclerosis in ApoE knockout mice [53], and Helkkula *et al*. also found that a PTV of ANGPTL8 can significantly reduce circulating TG levels in Finnish population and is associated with a lower incidence of CAD [23]. Interestingly, another 8-year follow-up study of 533 CAD patients found that high ANGPTL8 (upper tertile) was significantly negatively correlated with cardiovascular events, and the survival rate of high ANGPTL8 level group was much higher than that of medium/low-level groups [24].

### 2.5. Other metabolic diseases

In other metabolic diseases, the circulation level of ANGPTL8 also changed to some extent. In patients with NAFLD, sleep apnea syndrome (SAS), metabolic syndrome and PCOS, circulating ANGPTL8 levels were significantly increased [12,13,48,54]. Previous studies of our group showed that circulating ANGPTL8 levels decreased in patients with hyperthyroidism (such as Graves's disease) [55], while increased in patients with subclinical and clinical hypothyroidism [8,56]. Although GDM is accompanied by the increase of circulating ANGPTL8 level [57], and ANGPTL8 is also considered as a molecular marker for early prediction and prognosis of GDM [58,59], but during normal pregnancy, the circulating ANGPTL8 concentration decreases continuously even if insulin resistance and TG levels increase with the progress of pregnancy [60]. In general, ANGPTL8 is significantly associated with a variety of metabolically related diseases (or physiological states), which has also been confirmed by some prospective studies as a molecular marker to assist in diagnosis or prognosis. Of course, there are some controversies between the results of different studies. These controversies emphasize the importance of exploring the physiological mechanism of ANGPTL8.

## 3. Regulations of ANGPTL8

### 3.1. Hormonal and nutrient regulation of ANGPTL8

As another name RIFL (refeeding induced in fat and liver) shows, the physiological regulation of ANGPTL8 is significantly regulated by the fasting/refeeding signal [61]. Besides, the increased expression of ANGPTL8 in insulin resistance model or T2DM suggests that insulin signalling also plays a regulatory role in ANGPTL8 [9]. Although the earliest study observed that the expression of ANGPTL8 increased in the state of insulin resistance, with the progress of follow-up studies, people realized that insulin, rather than insulin resistance, promoted the expression of ANGPTL8 [7]. In the insulin resistance model of L02 hepatocytes induced by various factors (such as palmitic acid, dexamethasone, TNF-α, IL-1β and insulin), the expression of ANGPTL8 increased only in the presence of hyperinsulinaemia [7]. More interestingly, when the cells were cultured with insulin combined with another IR inducer, the induction effect of insulin on ANGPTL8 was counteracted. In fact, the expression of ANGPTL8 is related to PI3K/Akt pathway. That is to say, insulin acts on insulin receptor to activate PI3K/Akt pathway, and then the expression of ANGPTL8 increases; in insulin resistance, PI3K/Akt pathway was damaged and ANGPTL8 expression was downregulated. Similarly, PI3K/Akt inhibitors (LY294002 or MK-2206) completely blocked the induction of ANGPTL8 expression by insulin [7]. In addition, Zhang *et al*. showed that glucose combined with insulin can further increase the expression of ANGPTL8 in adipocytes [62]. These findings establish a link between refeeding signal and ANGPTL8 expression regulation; that is, food intake increases blood glucose and insulin levels, thus stimulating ANGPTL8 expression and secretion. Similar to insulin, another insulin receptor agonist, IGF-1 can also promote the expression of ANGPTL8 in a dose-dependent manner [7].

Besides insulin receptor, GLP-1 receptor can also activate PI3K/Akt signalling pathway and improve insulin resistance. GLP-1 receptor agonists (exenatide, liraglutide or GLP-1 (7-36) amide) stimulated the expression and secretion of ANGPTL8 in HepG2 cells in a dose-dependent manner, and increased the concentration of ANGPTL8 in the cell supernatant. The promotion of ANGPTL8 is mediated by GLP-1 receptor and its downstream PI3K/Akt signalling pathway, as GLP-1 (9-36) amide, the degradation product of GLP-1 (7-36) amide, or the addition of PI3K inhibitor (LY294002), can not promote ANGPTL8. Consistent with this, clinical data showed that after 16 weeks of GLP-1 receptor agonist treatment, circulating ANGPTL8 levels in T2DM patients were significantly higher than baseline levels [5]. Surprisingly, the increase in ANGPTL8 did not appear to be associated with improvements in body weight, fasting blood glucose and HbA1c [5].

### 3.2. Transcriptional regulation of ANGPTL8 expression

Research showed that the half-life of ANGPTL8 mRNA was only about 15.71 min, while ANGPTL8 protein was relatively stable, with a half-life of about 2.47 h [4]. Therefore, transcriptional regulation may play an important role in the circulation of ANGPTL8. A strong CCAAT/enhancer binding protein beta (C/EBPβ) co-binding site was found near −527 bp upstream of ANGPTL8 gene. Insulin treatment could significantly enhance the mRNA expression of C/EBPβ. After C/EBPβ silencing, the induction of insulin on the expression of ANGPTL8 in H4IIE cells and 3T3-L1 cells was significantly inhibited. Therefore, C/EBPβ may mediate insulin regulation of ANGPTL8 gene transcription [63]. On the other hand, the deletion of −60 to −309 bp region of hepatocyte ANGPTL8 promoter significantly reduced the basic promoter activity. The computational motif search results showed that the −84/−68 bp region of the promoter was a potential hepatocyte nuclear factor 1α/1β (HNF-1α/β) binding sequence. In hepatocytes, the mutation of HNF-1 binding site significantly decreased the promoter activity, and in non-hepatocytes, the mutation hindered the activation of HNF-1 binding site after co-transfection. After refeeding, the expression of HNF-1α in the liver of mice increased rapidly, which was consistent with the expression level of ANGPTL8 protein. After HNF-1 silencing/knockout, the protein expression of ANGPTL8 was significantly decreased in Hepa 1–6 cells and primary mouse hepatocytes, which completely blocked the induction of ANGPTL8 expression by insulin. Electrophoretic mobility shift assays showed that HNF-1 was directly bound to ANGPTL8 promoter region. Chromatin immunoprecipitation (ChIP) analysis also confirmed the recruitment of endogenous HNF-1 by ANGPTL8 promoter region. Therefore, HNF-1α also mediates refeeding signalling and insulin regulation of ANGPTL8 transcription [64].

T0901317, a ligand of liver LXRα, can increase the expression of ANGPTL8 in HepG2 and primary hepatocytes in a time-dependent manner. Similarly, overexpression of LXRα in primary hepatocytes significantly increased the mRNA and protein levels of ANGPTL8, while LXRα knockout significantly decreased the expression of ANGPTL8 in mouse liver. The upregulation of ANGPTL8 in liver and WAT induced by refeeding was significantly inhibited by blocking LXRα activation with GSK2033. Therefore, the upregulation of ANGPTL8 induced by refeeding is at least partially mediated by LXRα [4]. The binding site of carbon responsive element binding protein (ChREBP) was found in the promoter sequence of ANGPTL8, which provides a more specific mechanism for LXR to participate in the regulation of ANGPTL8 expression [25]. On the other hand, another downstream element of LXR, sterol regulatory element binding protein-1c (SREBP-1c), may also be involved in the regulation of liver ANGPTL8 expression. Saturated fatty acids and endoplasmic reticulum stress induced liver LXR expression, and combined with SREBP-1c promoter, induced SREBP-1c expression upregulated, thereby inducing fat synthesis, accompanied by the increase of liver ANGPTL8 expression level, while SREBP-1c siRNA transfection downregulated ANGPTL8 expression [65]. It is worth noting that SREBP-1c and peroxisome proliferator-activated receptor type-alpha (PPARα) signal have opposite expression trends in the regulation of liver lipid metabolism in obese patients. There was a competitive relationship between PPARα and LXR for antagonistic partner 9-cis retinoic acid receptor (RXR). PPARα phosphorylation induced by AMPK signal could significantly downregulate SREBP-1c expression induced by LXR [66–68]. The activation of AMPK decreased in the liver of obese mice, resulting in the damage of fatty acid β-oxidation pathway, while the activation of AMPK/PPARα pathway can improve obesity and fatty liver [69,70]. In contrast to the effect of LXR/SREBP-1c signal on the expression of ANGPTL8, AMPK agonists (AICAR and metformin) significantly inhibited the expression of ANGPTL8 in liver by activating AMPK/PPARα signal [65]. Therefore, the elevated level of ANGPTL8 in obese patients may be the result of the inhibition of AMPK signal and the enhancement of LXR/SREBP-1c signal.

On the other hand, the downregulation of ANGPTL8 expression during fasting may be related to glucocorticoid signalling. Dexamethasone significantly inhibited the expression of ANGPTL8, while mifepristone (RU486), a glucocorticoid signal inhibitor, significantly enhanced the expression of ANGPTL8. Liver-specific knockout of glucocorticoid receptor (GR) gene resulted in a significant decrease in the downregulation of ANGPTL8 expression induced by fasting. Further studies showed that there was a negative glucocorticoid response element (NGRE) palindrome sequence (CCTCNNGGAG) between −1096 and −1105 bp upstream of the transcription initiation site (TSS) of ANGPTL8 gene. ChIP analysis showed that GR was dynamically recruited to NGRE site in liver ANGPTL8 promoter region, which may mediate the inhibitory effect of glucocorticoid on ANGPTL8 expression [4].

In addition, vitamin D receptor (VDR) signalling may be involved in the regulation of liver ANGPTL8 expression. In HepG2 and uocyte liver cells transfected with adenovirus overexpressing human VDR gene, the mRNA of ANGPTL8 increased about 15-fold after vitamin D intervention for 4 h. The expression of ANGPTL8 was also increased after the lithocholic acid (LCA) intervention. Nevertheless, compared with vitamin D, the peak of ANGPTL8 expression (25-fold) was about 24 h after LCA intervention. However, vitamin D combined with LCA could further increase the expression level of ANGPTL8 (38-fold) [6]. VDR is considered to be related to non-alcoholic fatty liver (NAFL), although the specific mechanism is not fully clear [71,72]. In NAFL patients, the increased expression and signal intensity of VDR in liver partly explain the increased transcription level of ANGPTL8 in liver and the increased concentration of circulating ANGPTL8 protein. Elevated circulating FFA and hyperinsulinaemia are common features of NAFL. In a variety of liver cells, FFA and insulin have been proved to stimulate the expression of VDR, accompanied by increased expression of ANGPTL8 and accumulation of intracellular TG. In turn, ANGPTL8 knockdown significantly reduced FFA induced TG accumulation [6].

**Figure 1.**
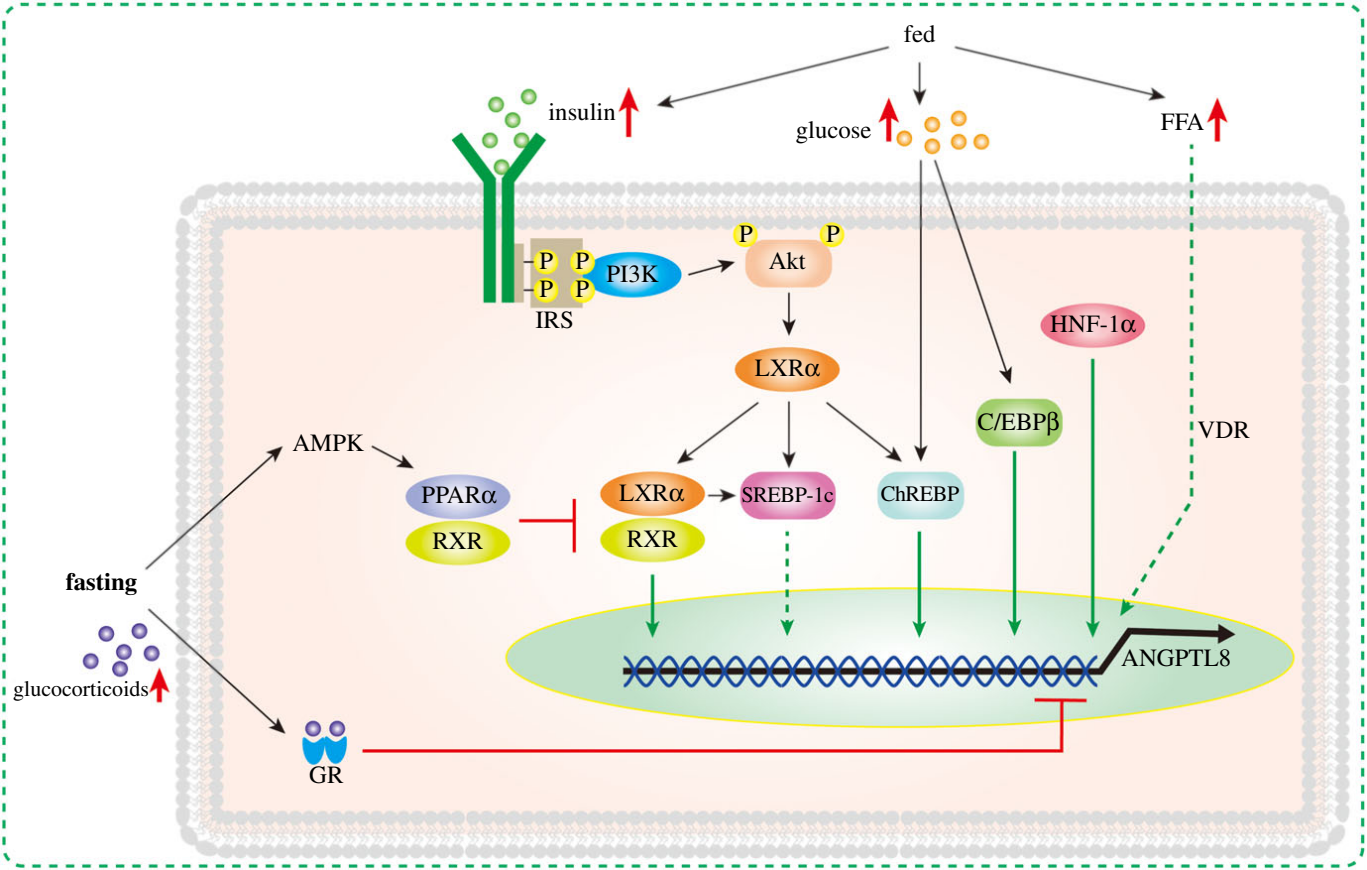
Regulation of ANGPTL8 expression during fasting/fed. Under feeding conditions, the increase of insulin, glucose and FA can increase the expression of ANGPTL8 through a variety of pathways. PI3K/Akt, LXRα, ChREBP, SREBP-1c, C/REBPβ, HNF-1α and VDR may participate in this process. Under fasting condition, GR signal inhibited the transcription of ANGPTL8. At the same time, PPARα competes with LXR to bind to RXR, which inhibits the induction of ANGPTL8 expression by LXR.

In general, a variety of nutrition and hormone-related signals are related to the regulation of ANGPTL8, and the current research may only explain part of them (figure 1). Insulin resistance is a typical feature of T2DM, accompanied by dyslipidaemia and hyperinsulinaemia. In most studies, the circulating level of ANGPTL8 increased under the condition of T2DM, but some studies showed that the circulating level of ANGPTL8 had no difference or even decreased compared with the control group. Similarly, there are such contradictory results and disputes in other metabolic diseases. Although the different kits may be one of the causes of these differences, the specific metabolic characteristics of the subjects, such as the intensity of PI3K/Akt signal and the distribution of lipids, should also be considered.

## 4. ANGPTL8 and glucose metabolism

Compared with the role of ANGPTL8 in lipid metabolism, its effect on insulin sensitivity and glucose metabolism has been controversial. Among them, the effect of changed ANGPTL8 expression on liver glucose production and overall blood glucose is the main controversial point. First, let us review the studies that support the beneficial effects of ANGPTL8 on glucose metabolism. In T2DM patients with insulin resistance, the regulation of insulin on HGP is defective. This is mainly reflected in two aspects: the inhibition of glycogen decomposition, and gluconeogenesis being weakened [73]. Guo *et al*. detected insulin signalling pathway related genes (IRS-1, Akt, JNK, mTOR, GSK3 β, FoxO1) and their respective phosphorylation levels in HepG2 cells overexpressing ANGPTL8 gene. The results showed that overexpression of ANGPTL8 increased insulin stimulated phosphorylation levels of Akt (ser473), GSK3 β (ser9), FoxO1 (ser256) in HepG2 cells. The activation of Akt/GSK3β and Akt/FoxO1 pathway plays an important role in promoting hepatic glycogen synthesis and inhibiting gluconeogenesis, respectively. Therefore, ANGPTL8 may be involved in the regulation of hepatic glucose metabolism. In accordance with this, the intracellular PAS staining showed more glycogen accumulation, and decreased expression levels of PEPCK and G6Pase after ANGPTL8 overexpression. Correspondingly, ANGPTL8 knockout resulted in decreased phosphorylation of Akt/GSK3β and Akt/FoxO1 pathways, decreased glycogen synthesis and increased gluconeogenesis in HepG2 [74]. In primary mouse hepatocytes, the overexpression of ANGPTL8 had no effect on Akt upon the inhibition of PI3K phosphorylation induced by LY294002, but it could counteract the inhibition of PI3K phosphorylation and the inhibition of Akt by MK-2206 (Akt inhibitor), thus preserving the activity of Akt and its downstream molecular pathway [18]. Previous studies have found that there are two casein kinase phosphorylation sites (Ser94 and Thr98) in ANGPTL8 protein, which may be related to protein phosphorylation [75]. The Ser94 or Thr98 mutation of ANGPTL8 reduced the insulin-induced Akt phosphorylation level, which was more effective in the case of double mutation [74]. Therefore, the protective effect of ANGPTL8 on insulin sensitivity is mediated by its direct regulation of Akt phosphorylation. Consistent with the results at the cellular level, the overexpression of ANGPTL8, through tail intravenous injection of adeno-associated virus, significantly improved the glucose tolerance and insulin sensitivity of mice in the glucose-tolerance and insulin-tolerance experiments [18]. On the other hand, the exocrine capacity of ANGPTL8 depends on its signal peptide sequence (1–20AA), and the deletion of this sequence has no effect on insulin-induced Akt phosphorylation, indicating that the ANGPTL8 protein acting on Akt mainly comes from liver cells itself [18].

However, inconsistent with the results of ANGPTL8 overexpression, Wang *et al*. showed that glucose homeostasis was not affected after ANGPTL8 knockout through homologous recombination using VelociGene technology in mice. There was no difference in fasting blood glucose and insulin levels between ANGPTL8 knockout mice and wild-type mice. In insulin-tolerance and pyruvate-tolerance tests, these two kinds of mice also showed similar blood glucose levels, indicating that ANGPTL8 knockout had no significant effect on insulin sensitivity and gluconeogenesis in mice [76]. Another study from Vatner *et al*. [77] showed that ANGPTL8 antisense oligonucleotide (ASO) could protect high-fat fed rats from glucose intolerance. However, in this study, ANGPTL8 ASO treatment resulted in increased TG uptake in WAT of rats, accompanied by increased adipose tissue mass [77]. This is inconsistent with the decrease of TG uptake in white adipose tissue (WAT, increased cardiac TG uptake) caused by ANGPTL8 knockout in the research of Wang *et al*. [76]. We noticed that Vatner *et al*. [77] introduced ASO into rats by intraperitoneal injection, resulting in a 73% decrease in ANGPTL8 expression in epididymal fat pad and an 82% decrease in liver during fasting. The difference of gene knockout/inhibition caused by different experimental methods may partly explain the difference of results. However, it is worth noting that the results of Vatner *et al*. also show a relationship between the increased TG uptake in WAT and the improvement of glucose metabolism [77]. This is consistent with the results from Zhang *et al*. study to a certain extent, that overexpression of ANGPTL8 leads to weight gain accompanied by improvement of glucose tolerance [18]. The improvement on glucose metabolism of ANGPTL8 is often accompanied by weight gain, which seems to imply that ANGPTL8 may affect the choice of energy substrates, so as to improve the global metabolism. In the following section on lipid metabolism we will introduce this point in detail.

Considering the complex relationship between glucose and lipid metabolism, the effect of ANGPTL8 on glucose metabolism is likely to be weakened and masked by its effect on lipid metabolism. In fact, although there was no significant difference in glucose homeostasis, the circulating TG and secretion of liver VLDL in ANGPTL8 knockout mice decreased significantly in the study of Wang *et al*. [76]. The overexpression of ANGPTL8 at cell level and animal level showed that ANGPTL8 could improve insulin sensitivity and inhibit HGP. Although further experiments are still needed, the regulatory effect of ANGPTL8 on glucose metabolism should exist.

## 5. ANGPTL8 and lipid metabolism

Before discussing the roles of ANGPTL8 in lipid metabolism, it is necessary to briefly describe absorption, transport and utilization of lipid, as well as ‘lipotoxicity’, although these have been covered in detail in previous reviews. Furthermore, some investigators have argued that adipose tissue, although somewhat reminiscent of obesity, may in fact be metabolically beneficial by secreting a number of cytokines, or acting as a ‘buffering pool’, that protects the organism from ‘lipotoxicity’.

### 5.1. Lipotoxicity

After being absorbed by intestinal epithelial cells, dietary fatty acids are assembled into TG and absorbed into the blood through chylomicron (CM) [78]. The uptake of circulating TG by peripheral tissues (fat, muscle, etc.) is mainly dependent on the LPL in capillaries, which hydrolyses TG to FA and glycerol, and then FA enters local cells for oxidation and energy supply, or transforms into TG again and stores it in lipid droplets (LDs). Although hypertriglyceridaemia is significantly associated with atherosclerosis and obesity is considered to be an important risk factor for metabolic diseases such as T2DM, the damage caused by lipid metabolism disorder is directly mediated by FAs. The increase of the concentration of NEFAs destroys the integrity of the biological membrane, changes the acid-base homeostasis of cells, and causes the production of harmful bioactive lipids. These effects in turn damage cell membrane function and induce endoplasmic reticulum stress, mitochondrial dysfunction, inflammation and cell death. In general, these harmful effects are collectively referred to as ‘lipotoxicity’ [79]. Most of cells can use glycerol to esterify FA to form inert TGs, which is particularly important for reducing fat toxicity [80]. Excessive TG hydrolysis can lead to insulin resistance. TG hydrolysates (DAG, FA, etc.) can damage insulin signalling by promoting the synthesis of toxic lipids such as ceramide and inducing oxidative stress [81–83]. In addition, lipolysis products can promote hepatic gluconeogenesis. Glycerol can be used as a direct raw material for gluconeogenesis [84]; fatty acids are metabolized to acetyl CoA after being transferred to the liver, which can enhance the activity of pyruvate carboxylase, thereby enhancing the entry of pyruvate into the gluconeogenesis pathway [85]. On the contrary, insulin can inhibit the activity of HSL, the rate limiting enzyme of lipolysis [86], and the occurrence of insulin resistance can lead to the enhancement of lipolysis and the increase of the release of lipolysis products, which in turn leads to the continuous deterioration of insulin resistance [87].

### 5.2. Metabolic benefits of adipose tissue

In fact, ectopic lipid accumulation can lead to fatty liver and insulin resistance, and adipose tissue, as the main TG storage site, can effectively isolate the toxic effect of lipid on the body [88]. Studies have shown that increasing the expression of key enzymes of fat synthesis in white fat can not only increase fat content, but also significantly improve insulin sensitivity [89]. In a high-carbohydrate environment, the body converts excess glucose into fat and stores it in adipose tissue, which is very important to ensure the stability of blood glucose. Insulin plays an important regulatory role in this process. On the one hand, insulin promotes the activation of mTORC1 through the PI3K/Akt pathway, stimulates the expression of SREBP-1c, and further activates the expression of various fatty acids and TG synthesis-related genes [90]. On the other hand, PI3K/Akt pathway can also increase the availability of precursors of adipogenesis (pyruvate, NADPH, etc.) and enhance the process of lipid synthesis by promoting GCK catalysed glycolysis [91]. In addition, insulin plays an important role in the regulation of GLUT4, especially in the uptake of glucose by adipocytes. Overexpression of GLUT4 in adipose tissue can effectively reduce fasting blood glucose and improve glucose tolerance, which is largely due to ChREBP induced glucose-dependent adipogenesis in WAT [92]. Last but not least, tissue variability in regulation of LPL activity by insulin also partially supports the metabolic benefits of adipocytes and highlights the impact of tissue variability of regulation of LPL on overall metabolism [93]. Therefore, WAT can be used as an effective buffer tissue to maintain energy balance in the face of high nutritional substrate.

### 5.3. Effects of ANGPTL8 on lipid metabolism

Although the other biological functions of ANGPTL8 are more or less controversial, its inhibitory effect on LPL has been recognized by most researchers. Many studies have confirmed that overexpression of ANGPTL8 can lead to impaired clearance of circulating TG, leading to hypertriglyceridaemia [53], while knockdown or inhibition of ANGPTL8 can significantly reduce the level of circulating TG [76,94,95]. Interestingly, after ANGPTL8 knockout, although LPL activity was enhanced, the uptake of VLDL-TG by WAT was decreased; on the contrary, the uptake of VLDL-TG by heart tissue was relatively increased [76]. This suggests that ANGPTL8 may play a directing role in the transport of postprandial TG to ensure that more energy substrates can be stored in WAT. ANGPTL8 knockout resulted in decreased tissue-specific TG uptake and reduced fat accumulation in adipose tissue. Consistently, ANGPTL8 knockout mice showed slow weight gain [76].

**Figure 2.**
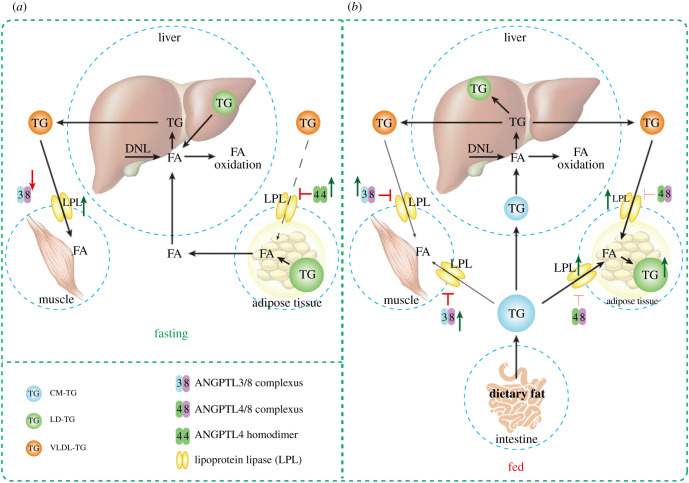
The directing effect of ANGPTL8 on TG during fasting/fed. (*a*) Under the fasting condition, the expression of ANGPTL4 increased, while the expression of ANGPTL8 decreased in WAT, which enhanced the inhibition of LPL activity and decreased the uptake of TG by WAT. At the same time, the active lipolysis in WAT results in the release of FA. The decrease of circulating ANGPTL3/8 complex alleviated the inhibition of LPL activity in muscle tissue, and the muscle obtained more energy substrates. (*b*) Under the feeding condition, the circulating ANGPTL3/8 complex increased and the local LPL activity in muscle tissue was significantly inhibited. At the same time, the expression of ANGPTL4 decreased in WAT, and the formation of ANGPTL3/4 complex weakened the inhibitory effect of ANGPTL4 on LPL. As a result, more TG is directed into WAT for storage.

In order to explain this interesting phenomenon, we need to introduce the other two members of the ANGPTLs family (ANGPTL3 and ANGPTL4). ANGPTL3, ANGPTL4 and ANGPTL8 all inhibited LPL activity and responded to fasting/refeeding signal. However, there are differences in tissue expression specificity, response to nutritional signals and inhibition of LPL among the three. (1) The expression of ANGPTL3 was the highest in liver, ANGPTL4 was mainly expressed in WAT, and ANGPTL8 was highly expressed in liver and WAT [61,96–98]. (2) For the inhibition of LPL, the ability from strong to weak was ANGPTL4, ANGPTL3 and ANGPTL8. It is worth noting that ANGPTL8 can bind to ANGPTL3 or ANGPTL4, which can enhance/weaken their inhibition of LPL respectively [16,99]. (3) Refeeding signal significantly increased the expression of ANGPTL8 (about 58-fold), but inhibited the expression of ANGPTL4 (about 40-fold); this may be due to the much higher expression of ANGPTL3 at baseline level than that of ANGPTL8, refeeding signal can only slightly induce the expression of ANGPTL3 (about 3-fold) [100], and some researchers believe that ANGPTL3 has no response to nutrition signal [101]. In conclusion, a possible model of action was established (figure 2): Under feeding conditions, the expression of ANGPTL8 in liver and WAT was significantly increased, while the expression of ANGPTL4 in WAT was significantly decreased. The decrease of ANGPTL4 and the formation of ANGPTL4/8 complex led to the increase of LPL activity in WAT, and more TG was transferred into adipose tissue for storage; in liver, the formation of ANGPTL3/8 complex increased and secreted into circulation, resulting in the inhibition of LPL activity in other parts and decreased TG uptake. The final result of this linkage effect is to ensure that postprandial TG can be stored more in WAT. Under the fasting condition, the expression of ANGPTL4 in WAT increased, and with the decrease of circulating/local ANGPTL8 level, the activity of LPL in WAT was significantly inhibited, and the output of TG from liver could be more directed to skeletal muscle and other tissues for oxidative energy supply. This reminds us of a previous experiment conducted by Farese *et al*. [93] in eight human subjects, in which LPL activity in adipose tissue was significantly increased, while that in skeletal muscle was decreased compared with baseline level 6 h after insulin/glucose infusion. Farese *et al*. speculated that this tissue-specific regulatory function of LPL would guide lipoprotein TG derived fatty acids away from muscle and into adipose tissue for storage [93]. In the previous studies of other researchers and review of our group, we have proposed that ANGPTL8 could guide the flow of postprandial TG by interacting with ANGPTL3 and ANGPTL4, and discussed its theoretical feasibility [17]. Following this, Chen *et al*. provided direct and more detailed experimental data, proving the existence of this action model [100]. Although the inhibition of ANGPTL4 can also enhance the local LPL activity of adipose tissue in theory, the excess lipid can be directed to adipose tissue. As found in the study of Aryal *et al*. [102], the loss of adipose tissue-specific ANGPTL4 can prevent excessive ectopic lipid deposition in liver and muscle, reduce the translocation of new PKC (nPKC) membrane and enhance insulin signal transduction. However, previous studies have shown that ANGPTL4 knockout leads to severe acute phase reactions [103], which challenge the safety of targeting ANGPTL4 in the treatment of glucose and lipid metabolism disorders. By contrast, it seems to be a more appropriate attempt to enhance the ‘metabolic switch’ effect of ANGPTL8.

In addition to inhibiting the activity of LPL and thus reducing the cyclic TG hydrolysis, ANGPTL8 also plays a regulatory role in intracellular TG hydrolysis. Intracellular TG hydrolysis is the process of decomposing TG in LDs into fatty acids for export (adipose tissue) or energy supply (non-adipose tissue), including neutral hydrolysis and acidic hydrolysis. Neutral hydrolysis is divided into three steps, which are dependent on adipose triglyceride lipase (ATGL), hormone-sensitive lipase (HSL) and MG lipase (MGL) [80]. In 3T3-L1 cells, the loss of ANGPTL8 resulted in an 18–19% decrease in TG storage and a significant increase in the release of non-esterified fatty acids (NEFAs). In accordance with this, the expression of other genes related to lipolysis and fatty acid oxidation was also significantly upregulated. This suggests that ANGPTL8 signalling in adipocytes may inhibit TG hydrolysis and subsequent fatty acid oxidation [104]. The regulatory effect of ANGPTL8 on intracellular TG neutral hydrolysis seems to be related to ATGL. In liver cells, the intervention of recombinant ANGPTL8 protein significantly downregulated the expression of ATGL [105]. In line with its inhibitory effect on intracellular TG hydrolysis, the overexpression experiment showed that ANGPTL8 could promote the differentiation of bovine adipocytes and the formation of LDs, which was consistent with the effect of circulating ANGPTL8 on postprandial TG directed to WAT, that is, ANGPTL8 increased the storage of energy substrates in WAT [106]. On the contrary, inhibition of ANGPTL8 resulted in hypermetabolism of white adipocytes, characterized by increased mitochondrial content, mitochondrial biomarkers and beige adipocytes [107]. Similarly, combined knockout (or antibody blocking) of ANGPTL8 and ANGPTL3 can also make mice show a hypermetabolic phenotype [108]. Blocking AMPK signalling pathway can eliminate the beiging characteristics caused by ANGPTL8 knockdown, which indicates that the downregulation of ANGPTL8 may activate AMPK signalling pathway, resulting in browning of white adipocytes [107]. However, interestingly, although the expression of ANGPTL8 in BAT is higher than that in WAT, the increased thermogenesis caused by the combined knockout of ANGPTL3 and ANGPTL8 does not occur in BAT and epididymal adipose tissue, and the difference of TG storage in different adipose tissue is a possible explanation [108].

**Figure 3.**
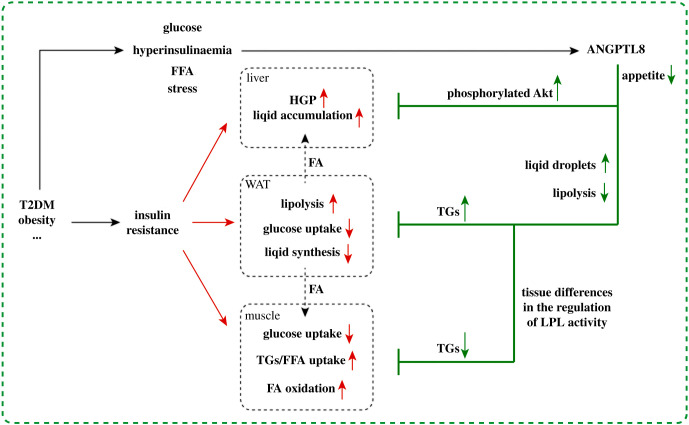
Compensatory increase of ANGPTL8 in pathological state improves metabolism. Insulin resistance occurs in the pathological state of diabetes and obesity, which leads to a variety of metabolic disorders in liver, fat and muscle. Hyperinsulinaemia, glucose, FA and stress induced compensatory increase of ANGPTL8. ANGPTL8 not only improves insulin sensitivity by enhancing Akt phosphorylation, but also enhances the storage and buffering capacity of WAT, reducing the damage of ectopic lipid pile and lipid toxicity in other tissues.

The role of ANGPTL8 in postprandial TG targeting and lipid uptake and storage in WAT has been confirmed in many studies, but its effect on de novo lipid synthesis (DNL) in adipose tissue has not been fully reported. Dang *et al*. showed that overexpression of ANGPTL8 significantly increased the expression of NEFAs uptake, FA synthesis and VLDL secretion related genes in the liver, accompanied by increased VLDL secretion [4]. Knockout of ANGPTL8 resulted in decreased liver VLDL secretion. This seems to indicate that ANGPTL8 in hepatocytes has a regulatory effect on the lipid output of liver. On the other hand, ANGPTL8 is associated with the improvement of insulin sensitivity, possibly through the regulation of Akt phosphorylation and inhibition HGP [18]. At the same time, the expression of ANGPTL8 is regulated by insulin and nutritional status *in vivo*, in which SREBP-1c, ChREBP and other regulatory elements/factors related to fat synthesis are involved [25]. Therefore, ANGPTL8 may mediate or promote the regulation of insulin on fatty acid synthesis, which also improves glucose metabolism to a certain extent. It should be pointed out that the decrease of HGP and the increase of lipid synthesis are coordinated with the function of ANGPTL8 in promoting lipid uptake and storage of WAT (figure 3).

## 6. Other physiological and pathological significances of ANGPTL8

Early studies have suggested that ANGPTL8 can promote the proliferation of islet β cells, which is the origin of another name: betatrophin. However, a number of subsequent studies have denied this result [109], so this review will not introduce this part. On the other hand, ANGPTL8 is also involved in the regulation of appetite, that is, the increase of ANGPTL8 is related to the occurrence of anorexia, and neuropeptide Y-positive neurons in the dorsal medial hypothalamic nucleus may be involved in it [110,111]. The increased expression of ANGPTL8 caused by refeeding may feedback prevent the excessive feeding behaviour of the body, which was introduced in our previous review, so this article will not elaborate on it [17].

## 7. Conclusion

ANGPTL8 is an important nutritional signal regulator, which is expressed in liver, fat and other tissues, and plays a regulatory role in postprandial lipid orientation. In pathological conditions such as T2DM, circulating hyperinsulinaemia stimulated the expression and secretion of ANGPTL8 [7]. Although current studies suggest that ANGPTL8 may be a harmful molecule; that is, elevated baseline ANGPTL8 levels are associated with a higher risk of retinopathy and all-cause mortality in patients with T2DM [20–22]. At the same time, inhibition of ANGPTL8 appears to be beneficial, manifested by a decrease in circulating triglycerides [112]. However, the following evidence cannot be ignored. (i) The directing function for circulating TG of ANGPTL8 emphasizes the buffering function of WAT, which can enhance the lipid storage of WAT and relatively reduce the lipid toxicity in other tissues [76,100]. (ii) Insulin and GLP-1 can promote the expression and secretion of ANGPTL8, while the increased ANGPTL8 can regulate Akt phosphorylation, which can directly improve insulin sensitivity [18]. (iii) Single-nucleotide mutation of ANGPTL8 has been proved to be associated with the risk of T2DM and cardiovascular disease. Bioinformatics analysis showed that Arg59Trp substitution caused by T allele, which has been shown to reduce the stability of ANGPTL8 protein, were associated with an increased risk of T2DM or impaired glucose tolerance [113–115]. (iv) Higher levels of ANGPTL8 in CAD patients are associated with lower incidence of cardiovascular events and predict higher long-term survival [24]. These results support the potential beneficial effect of ANGPTL8 on glucose and/or lipid metabolism. Therefore, we speculate that elevated circulating ANGPTL8 may have some beneficial effects on metabolic regulation, at least not completely harmful. Of course, in order to understand the role of ANGPTL8 in metabolic disorders, some suggestions must be made. First of all, a more reliable detection method is the basis. At present, many clinical studies use ELISA to detect the concentration of circulating protein, but the recognition sites of different kits are different. Secondly, dietary status at the time point of detection may have an impact on the results, considering the significant regulatory role of nutritional signals on ANGPTL8. Moreover, ANGPTL8 does not work in isolation, and ANGPTL3 and ANGPTL4 must be considered at the same time. Finally, it should be pointed out that the effects of ANGPTL8 on glucose metabolism and lipid metabolism cannot be treated separately, given the close and complex relationship between them. At the same time, the storage capacity of adipose tissue is limited. Whether the lipid storage exceeds the ‘critical value’ is very important for the metabolic benefits of adipose tissue.

## Data accessibility

This article has no additional data.
